# HEB in the Spotlight: Transcriptional Regulation of T-Cell Specification, Commitment, and Developmental Plasticity

**DOI:** 10.1155/2012/678705

**Published:** 2012-04-22

**Authors:** Marsela Braunstein, Michele K. Anderson

**Affiliations:** Sunnybrook Research Institute, University of Toronto, 2075 Bayview Avenue, Toronto, ON, Canada M4N 3M5

## Abstract

The development of T cells from multipotent progenitors in the thymus occurs by cascades of interactions between signaling molecules and transcription factors, resulting in the loss of alternative lineage potential and the acquisition of the T-cell functional identity. These processes require Notch signaling and the activity of GATA3, TCF1, Bcl11b, and the E-proteins HEB and E2A. We have shown that HEB factors are required to inhibit the thymic NK cell fate and that HEBAlt allows the passage of T-cell precursors from the DN to DP stage but is insufficient for suppression of the NK cell lineage choice. HEB factors are also required to enforce the death of cells that have not rearranged their TCR genes. The synergistic interactions between Notch1, HEBAlt, HEBCan, GATA3, and TCF1 are presented in a gene network model, and the influence of thymic stromal architecture on lineage choice in the thymus is discussed.

## 1. T-Cell Progenitors and Lineage Plasticity

During hematopoiesis, pluripotent progenitors are sequentially restricted in lineage potential and progressively committed to a single lineage choice. Lineage commitment is, therefore, established in part by the inability to respond to environmental cues, migrate to inductive environments, and/or express key lineage regulatory factors that direct the acquisition of alternative fate choices [1]. However, the thymus, a site where T cells are generated, does not produce stem cells, and the generation of T cells depends solely on the intermittent input of progenitors from adult bone marrow [2]. Circulating progenitors such as lymphoid-primed multipotent progenitors (LMPPs) or common-lymphoid progenitors (CLPs) enter the thymus at the corticomedullary junction (CMJ). During development, T-cell progenitors transition through two functionally distinct zones of the thymus: immature cells migrate outward through the cortex, while the more mature cells migrate inward toward medulla [1]. The developmental status of thymocytes can be identified by their cell-surface marker expression. The most immature progenitors lack the expression of CD4 and CD8 (double negative, DN) and are further discriminated based on the expression of CD44 and CD25 into four sequential stages: DN1 (CD44^+^CD25^−^), DN2 (CD44^+^CD25^+^), DN3 (CD44^−^CD25^+^), and DN4 (CD44^−^CD25^−^) [3].

The DN1 population is quite heterogeneous and has the capacity to generate multiple lineages [4]. Since DN1a (c-kit^+^CD24^−^) and DN1b (c-kit^+^CD24^+^) cells generate T cells efficiently and exhibit a strong proliferative capacity, they are considered to be the canonical early T-cell progenitors (ETP). The remaining DN1 subsets, DN1c (c-kit^int^CD24^−^), DN1d (c-kit^−^CD24^+^), and DN1e (c-kit^−^CD24^−^), are noncanonical T-cell progenitors because they lack the proliferative potential and differ substantially in their capacity to generate T cells. The heterogeneity of the DN1 population reflects the variety of non-T-cell lineages that are generated in the thymus. While DN1c and DN1d cells give rise to B cells, DN1a, DN1b, and to a small degree DN1e cells can produce natural killer (NK) cells [4]. The DN1c, DN1d, and DN1e subsets have also been shown to have the potential to generate dendritic cells (DCs) in the thymus [5, 6]. In addition, ETPs can be further separated into two subsets based on the expression of Flt3; the Flt3^+^ ETPs can give rise to B cells, while Flt3^−^ ETPs no longer possess B-cell potential [7]. Lastly, ETPs have the potential to generate myeloid cells in the thymus [8]. These studies indicate that B-cell potential is lost before myeloid potential in T-cell precursors prior to T-lineage commitment.

## 2. T-Cell Development: Gene Specification, Commitment, and Developmental Checkpoints

Specification into the T-cell lineage occurs during the transition from the DN1 to the DN2 stage, when lymphoid- and T-lineage-specific genes are turned on [9]. Some of the most important targets of T-lineage regulators include *Rag* genes, interleukin 7 receptor *α* (*IL7R*α**), *lck*, *Bcl11b*, *pT*α**, and *CD3* genes. Based on the expression of lck and c-kit, DN2 cells can be further separated into DN2a (lck^−^, c-kit^hi^CD25^+^) and DN2b (lck^+^, c-kit^int^CD25^+^) subpopulations, which display differential lineage potential; while DN2a can give rise to myeloid, NK, and DC cells, DN2b are T-lineage restricted [10, 11]. However, the revised model of hematopoiesis, in which the lymphoid-myeloid segregation occurs after the T-B segregation [8], has been recently challenged by a study involving IL7R-reporter mice [12]. In this study, myeloid cells did not arise from the cells that had a history of IL7R expression as tracked by a fate-mapping reporter gene, even in the DN1a and DN1b fractions [12]. These results suggested that myeloid cells in the thymus may not share a common intrathymic precursor with T-cells. Additional studies are needed to resolve this issue.

T-lineage-restricted DN2b cells progress to the DN3 stage. At the DN3 stage, the *TCR*β** gene is rearranged and expressed. Successfully produced TCR*β* chains pair up with invariant pT*α* chains, and with the CD3 components into a pre-TCR complex. Signaling through the pre-TCR grants survival and differentiation to the DN4 stage. In addition, the cells turn off the expression of *Rag* genes in order to prevent rearrangement of a second TCR*β* allele, a process called allelic exclusion. Finally, the cells proliferate and differentiate into the DN4 stage. The overall process resulting in allelic exclusion as well as cellular survival, proliferation, and differentiation is referred to as *β*-selection and represents the first checkpoint in T-cell development [13]. This checkpoint ensures that cells lacking productive *TCR*β** genes do not proceed further in development. The cells that have not received a pre-TCR signal die by apoptosis, unless they were previously predisposed to differentiate into the *γδ* T-cell lineage by the expression of TCR*γ* and TCR*δ* chains. Interestingly, pre-TCR signaling has also been linked to the inhibition of the tumour suppressor gene, p53, which functions in response to DNA damage [14]. An accumulation of p53 causes a cell-cycle arrest by activation of cell-cycle inhibitor genes such as p21, to support DNA repair. Alternatively, unrepaired DNA damage can also cause p53-induced death by activation of proapoptotic molecules. The mechanisms that link pre-TCR signaling to p53 induction have yet to be established.

 Following *β*-selection, CD8 is upregulated slightly earlier than CD4 in mice, resulting in cells at the immature CD8 single positive (ISP) stage. ISP cells can be distinguished from the mature CD8^+^ single positive (SP) cells by lack of cell surface TCR*β*. As the cells progress into the CD4^+^CD8^+^ (double positive, DP) stage, the expression of *Rag* genes is reinstated and *TCR*α** gene rearrangements take place. TCR*α* chains pair up with the TCR*β* chains and the CD3 components to form the mature TCR*αβ* complex, which interacts with peptide-MHC complexes expressed by the thymic stromal cells or thymus-resident APCs. TCR interactions with MHC and self-peptide result in positive and negative selection of DP thymocytes, which represent a second checkpoint in T-cell development and result in the generation of mature CD4^+^ and CD8^+^ SP cells.

## 3. Critical Regulators of Early T-Cell Development

 Proper development of T cells depends on the timing and level of transcription of lineage-specific regulatory genes. During hematopoiesis, transcription factors coordinate complex developmental events by modulating an array of genes that reduce multilineage potential and steer development toward particular lineage fates [15]. The activity of the transcription factors depends on their dosage, availability of their partners, as well as their overall binding specificity and affinity for a consensus DNA sequence. Transcription factors that are important for T-cell specification and commitment include Notch/CSL, GATA-3, TCF1, Bcl11b, and E proteins.

### 3.1. Notch Signaling

 Notch signaling is an evolutionarily conserved mechanism that influences cell fate through cell-cell interactions. Notch proteins are transmembrane receptors that signal in a ligand-dependent manner. Flies have one Notch receptor, and two ligands: Serrate and Delta. Mammals, however, possess four Notch receptors (Notch1 to 4) and five ligands: two Serrate-like ligands called Jagged-1 and Jagged-2, and three Delta-like (DL) ligands called DL1, DL3, and DL4. Upon receptor-ligand engagement, a series of proteolytic cleavages take place that liberate the intracellular segment of Notch (ICN). ICN is the active form of Notch, which binds to CSL (CBF-1/RBP-J*κ* in mammals, Suppressor of Hairless in *Drosophila*, Lag-1 in *C. elegans*) displacing the Groucho corepressor and recruiting coactivators such as Mastermind to the complex. These events initiate transcription of Notch-target genes, such as *Hes1, Deltex1, CD25, pT*α**, and *TCR*β** [16].

**Figure 1:**
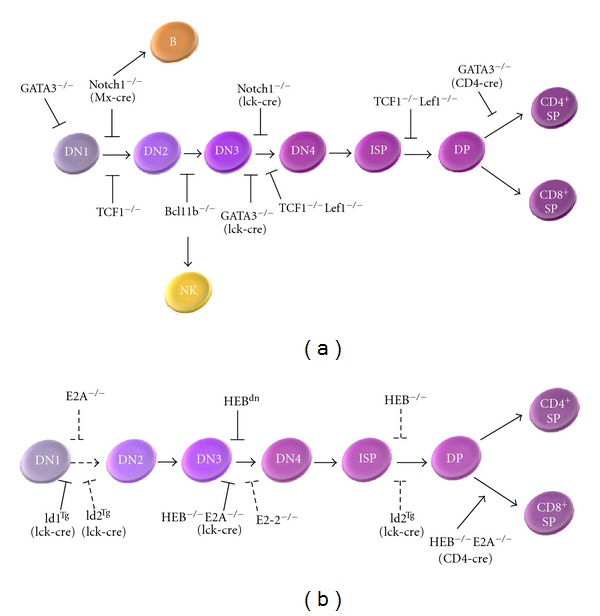
Key transcription factors in early T-cell development. Regulation of early T-cell development occurs through the coordinated action of transcription factors. (a) GATA3 is important for the generation of DN1 cells, and GATA3^−/−^ mice fail to produce any T-cells. Inactivation of GATA3 during DN stages results in a block at the DN3 stage due to defects in TCR*β* expression. When GATA is inactivated at the later stages of T-cell development, no CD4 SP cells can be generated. Notch1 signalling is indispensable for T-cell specification and commitment since mice deficient in Notch1 give rise to intrathymic B cells at the expense of T cells. Notch1 inactivation during DN stages arrests T-cell development at the DN3 stage due to the defects in V-(D)J rearrangements of the *TCR*β** locus. Inactivation of TCF1 and LEF1 simultaneously results in the partial block at the DN3 stage and a complete block at the ISP stage as cells fail to rearrange *TCR*α** locus. Lastly, Bcl11b is essential for the specification into the T-cell lineage. Bcl11b^−/−^ cells fail to progress past the DN2 stage of T-cell development, and instead, differentiate into NK cells. (b) T-cell blocks associated with mutations in HEB, E2A and/or E2-2 E-proteins and their antagonists, Id1 and Id2. Solid blunt lines indicate complete developmental arrest, while dotted blunt lines indicate a partial developmental arrest. DN: double negative, DP: double positive, SP: single positive, ISP: immature single positive, NK: natural killer, B: B lymphocytes.

 Among the four Notch receptors, Notch1 plays an indispensable role in T-cell development, particularly in the T/B lineage choice. Mice deficient for Notch1 in HSCs display an arrest at the DN1 stage of T-cell development and generate B cells intrathymically (Figure 1(a)) [17]. Furthermore, conditional inactivation of Notch1 at the DN stages has shown that Notch1 signaling is necessary for *TCR*β** rearrangement and for generation of *αβ* but not *γδ* T cells from DN3 progenitors [18–20]. Although thymocytes also express Notch2 and Notch3, mice deficient for either of these receptors do not have pronounced disturbances in T-cell development [21–23]. Likewise, Notch4-deficient mice do not have any detectable defects in T-cell development [21]. Interestingly, progenitors constitutively expressing ICN develop into T cells in bone-marrow at the expense of B cells [24], indicating that the bone marrow environment is well equipped to support T cell development apart from the lack of DL Notch ligands [25]. Although Notch1 receptor has the capacity to interact with either DL1 or DL4 [26], DL4 represents the primary physiological partner for Notch1 receptor in T-cell development [27, 28]. Thymic stroma, therefore, provides essential Notch ligands that are not expressed by the bone-marrow stroma, which helps to explain the unique capacity of the thymus to support T-cell development.

### 3.2. GATA3

 The GATA family includes three zinc-finger transcription factors, GATA1, GATA2, and GATA3, which bind to the consensus GATA motif in DNA. Within the hematopoietic system, all three GATA factors are expressed in the hematopoietic progenitors; however, GATA1 is also expressed in the cells of the myeloid origin, such as erythrocytes, mast cells, eosinophils, and megakaryocytes, while the expression of GATA2 is limited to mast cells and megakaryocytes [29]. GATA3, on the other hand, is most abundantly expressed in T cells and NK cells [30–32]. During T-cell development, the expression of GATA3 gradually increases from the DN1 to the DN3 stage then diminishes at the DN4 stage. GATA3 is repressed at the DP stage and becomes upregulated again in the CD4 SP cells, but it stays off in the CD8 SP cells [33].

GATA3^−/−^ mice die at E11 due to defects in the development of the central nervous system [34]. The essential role of GATA3 in the generation of T cells was revealed in experiments involving antisense oligos against GATA3 [35], and by generation and examination of blastocyst chimeras from GATA3^−/−^ and Rag-2^−/−^ embryonic stem cells (Figure 1(a)) [36]. GATA3 is also necessary for the generation of ETPs [32]. Conditional inactivation of GATA3 at the DN stage of T-cell development has revealed that GATA3 is also required for passage through *β*-selection and for the proper expression of TCR*β* protein [37]. Inactivation of GATA3 at the later stages of T-cell development has shown that GATA3 is also essential for the generation of CD4^+^ SP cells [37, 38]. GATA3 binds to the promoter regions and directly regulates the expression of other genes important for T-cell development, such as the *Rag* genes [39] and Th-POK, a CD4 cell specifying transcription factor [40]. Elevated levels of GATA3 in early T-cell development inhibit T-cell development by downregulating genes involved in T-cell specification [41].

### 3.3. TCF1

*Wnt* genes encode numerous Wnt factors, which are soluble glycoproteins secreted by thymic epithelial cells. Wnt factors provide intracellular signaling to different cell types, including developing thymocytes. Wnt-mediated signaling is initiated when Wnt binds to Frizzled receptors and the low-density lipoprotein receptor-related protein (LRP)-5 and LRP6 on the cell surface of developing thymocytes [42]. The signaling cascade stabilizes cytoplasmic *β*-catenin, which translocates into the nucleus and displaces a corepressor called Groucho from the T-cell factor 1 (TCF1) and the lymphoid enhancer factor 1 (LEF1) transcription factors. Stabilized *β*-catenin collaborates with pre-TCR signaling to ensure thymocyte survival [43, 44]. In the absence of Wnt, *β*-catenin is targeted for degradation by ubiquitination [45], thus leaving the TCF1/Groucho complex to function as a transcriptional repressor.

TCF1 and LEF1 share a homology domain with proteins of the high mobility group (HMG) family. The expression of TCF1 is restricted to T cells, with the highest expression occurring across the *β*-selection checkpoint and at the ISP stage of T-cell development [46]. T-cell development is impaired at multiple stages in TCF1-deficient mice (Figure 1(a)). First, there is a complete block at the DN1 stage when TCF1^−/−^ stem cells are cultured on OP9-DL4 stroma, which support T-cell development *in vitro *[47]. Second, in TCF1^−/−^ mice, there is a partial block at the DN1 to DN2 transition. Lastly, there is a marked accumulation of cells at the ISP stage and reduced overall numbers of thymocytes [46, 48, 49]. LEF1^−/−^ mice have many abnormalities, but none that are associated with thymopoiesis [50]. The potential redundancy between the two factors was tested by generating mice deficient in both TCF1 and LEF1 [50]. Indeed, T-cell development was partially blocked at the DN3 stage and completely blocked at the ISP stage in TCF1/LEF1^−/−^ mice due to the impaired expression of the *TCR*α** gene.

### 3.4. Bcl11b

Bcl11b (B-cell lymphoma/leukemia 11b) is a tumour suppressor gene that encodes for three zinc-finger transcription factors: *α*, *β*, and *γ*. Bcl11b*α* and *β* are expressed at high levels in the thymus, while the expression of *γ* is low [51]. The gene was discovered while studying the thymic lymphomas in mice with mutations or deletions in the Bcl11b gene locus [52]. A Bcl11b homologue exists called Bcl11a, which functions as an oncogene in certain B-cell leukemias that involve Ig heavy-chain gene translocations [53].

An appreciation for the importance of Bcl11b in T-cell development stemmed from studies involving Bcl11b knockout mice. Bcl11b^−/−^ thymocytes have severe defects in V-(D)J *TCR*β** gene rearrangements resulting in apoptosis and arrest at the ISP stage [51]. The timing of developmental arrest suggested that Bcl11b has a regulatory connection with TCF1, and recent evidence suggests that Bcl11b is a direct target of TCF1 [47]. Furthermore, conditional inactivation of Bcl11b at earlier stages of T-cell development revealed a block at the DN2 stage and an increased production of NK cells [54, 55]. Bcl11b, like TCF1, is directly upregulated by DL-Notch signaling, implicating it as a mediator of the impact of Notch1 on alternative lineage choice. These studies have identified Bcl11b as a critical factor for early T-cell development (Figure 1(a)).

### 3.5. E Proteins

E proteins belong to the class I basic helix-loop-helix (bHLH) family of transcription factors. They control a variety of developmental processes in vertebrates such as myogenesis, neurogenesis, pancreatic development, and lymphopoiesis [56]. All E proteins possess a stretch of basic amino acids capable of binding DNA. Furthermore, E proteins function as homodimers as well as heterodimers with other E proteins or HLH factors. The crystal structure of the bHLH domain revealed that each subunit of the dimer contacts one half of the E-box site [57]. The contact with DNA is established via the basic region, while the HLH domain participates in dimerization. Binding to DNA, however, is not sufficient to activate transcription; rather, E proteins possess one or two activation domains (AD1 and AD2) [58–60], which mediate transcription by recruiting coactivators or corepressors to the complex. A repressive function may be conferred on E proteins upon binding to ETO factors, whereas activation may be enhanced by recruiting p300 to the transcription complex [61]. These factors competitively bind to the AD1 domain, enabling context-dependent regulation of gene expression.

**Figure 2:**
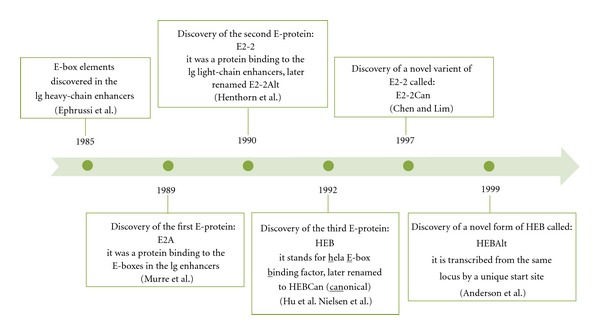
Timeline of E-protein discovery. In 1985, Ephrussi et al. identified regions in the immunoglobulin (Ig) heavy chain gene enhancer (*μ*E1–*μ*E5) that were occupied by unidentified DNA-binding proteins in the B-cell lines, but not in nonlymphoid cells [62]. The regions had a CANNTG consensus sequence, which was later named an E-box. In 1989, Murre et al. discovered that E-boxes in the Ig-heavy and light-chain enhancers were occupied by two novel proteins, which they named E47 and E12 [63]. These were the first E-proteins discovered, so called because they bind to the E-box sites. In 1990, the search for transcription factors that bind Ig light-chain enhancer sites (*κ*E1–*κ*E3) revealed a second E-protein, ITF-2A [64], later named E2-2Alt [65]. Concurrent studies by Hu et al. involved the use of the *μ*E2 sequence to screen a cDNA library from HeLa cells, a human cell line, which led to the discovery of the third E-protein in 1992 [66]; the mouse counterpart was discovered later that year [67]. This protein was named HEB (HeLa E-box binding factor). In 1997, a splice variant of E2-2 was identified [68] and named ITF-2b (now called E2-2Can), which in contrast to E2-2Alt had an inhibitory effect on the promoter of a muscle-specific gene [69]. In 1999, Anderson et al. set out to identify transcription factors involved in T-cell specification by screening a SCID (severe combined immunodeficient)-thymocyte cDNA library. The search revealed a novel HEB clone [70], which was transcribed from the HEB locus from its own transcriptional start site located near a unique alternative (Alt) exon, homologous to E2-2Alt [71]. The presence of the Alt exon resulted in naming this E-protein HEBAlt, and referring to the canonical HEB as HEBCan.

**Figure 3:**
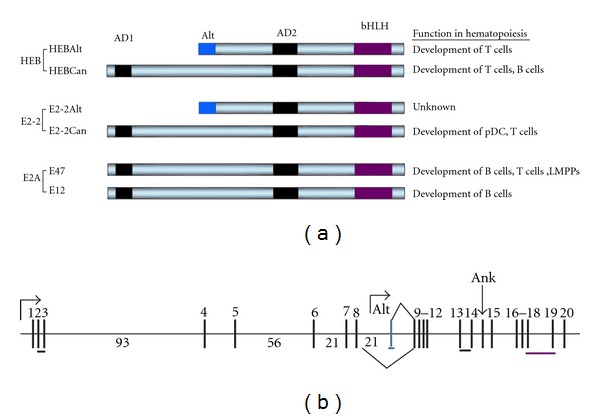
Structure of E-proteins. (a) E-proteins belong to the basic helix-loop-helix (bHLH) family of transcription factors. There are three genes, each encoding for two proteins: HEB (HEBCan and HEBAlt), E2-2 (E2-2Can and E2-2Alt), and E2A (E47 and E12). While E2A proteins are produced by alternative splicing, HEB and E2-2 factors are generated by independent transcription start sites and alternative splicing. All six transcription factors have a basic helix-loop-helix (bHLH) domain, which enables transcription factor dimerization and binding to the DNA. Activation domains 1 (AD1) and AD2 help recruit coactivators to the transcriptional complex. The Alt domain replaces AD1 found in the canonical forms of E-proteins and is conserved between mouse HEBAlt and E2-2Alt as well as through vertebrate evolution. (b) Organization of HEB gene. Vertical grey bars represent exons. Protein domains encoded by exons are shown as horizontal bars. Numbers above exons represent exon numbers, while numbers between exons indicate genomic distance in kb. pDC: plasmacytoid dendritic cells, LMPPs: lymphoid primed multipotent progenitors.

The E protein family is comprised of three members, *E2A*, *E2-2*, and *HEB*; the timeline of their discovery is outlined in Figure 2. E proteins are indispensable for the generation of LMPPs and HSCs, and for normal B-cell, T-cell, and plasmacytoid DC (pDC) development. Each gene encodes two proteins, as illustrated in Figure 3(a). The genes have alternative names as follows: E2A (aka TCF3 or ALF2), E2-2 (aka ITF2 or TCF4), HEB (aka TCF12, ALF1, or ME1). The *E2A* gene locus gives rise to E47 and E12 by alternative splicing [72]. The *HEB* gene locus on the other hand, has two transcription start sites which are responsible for generating the long form of HEB, called HEBCan, and the short form of HEB, called HEBAlt [71]. The *E2-2 *gene locus has the same type of genomic structure as the *HEB* gene locus, and also produces two forms, E2-2Can and E2-2Alt. As shown in Figure 3(b), the *HEB* gene locus is organized into 21 exons and spans a genomic area that is over 200 kb in size [71]. HEBCan is encoded by exons 2–20, and excludes the Alt exon by alternative splicing. The transcription of HEBAlt initiates just upstream of a unique Alt exon, and the transcript shares exons 9–20 with HEBCan. An ankyrin-like exon can be included in HEBCan but does not appear to be present in transcripts cloned from thymocyte cDNA libraries [70, 71]. The Alt exon encodes for a 23 amino acid Alt domain, which is 80% identical to the Alt domain of E2-2Alt. Amino acid alignment of Alt domains from HEBAlt cDNA from fish, chicken, mouse, and human revealed a high degree of identity, indicating that the Alt domain plays an important and conserved function in vertebrates.

## 4. Negative Regulation of E Protein Function

E proteins are expressed widely in mouse tissues. Their functions are negatively regulated by three mechanisms: through direct competition for the E box DNA binding sites, by posttranslational modifications, or through protein-protein interactions. The transcription factor ZEB has been shown to compete for the E-box binding sites within the Ig heavy-chain gene enhancer, thus inhibiting E protein activity in a cell-specific manner [73]. Posttranslational modification, such as ubiquitination of E2A proteins upon signaling through Notch1 receptor [74] or calmodulin-mediated inactivation of E2A [75], represents another potential mechanism by which E protein function is regulated. In addition, HEB-Tal1 heterodimers suppress expression of some HEB target genes through competitive binding to the E box sites [76]. Lastly, Id factors, which lack DNA-binding capacity, antagonize E protein function by forming stable inactive Id/E protein heterodimers [77]. This form of negative regulation seems to be the most well-understood mechanism by which E-protein function is regulated during T-cell development.

There are four mammalian Id factors, Id1, Id2, Id3, and Id4 [78], which vary in tissue distribution. Id1 and Id3 factors are widespread in adult and embryonic mouse tissues [65]. In contrast, Id2 transcripts are only detected in bone marrow, testes, and brain of adult mice and in fetal livers after 13.5 days of gestation [79]. Id4 is not expressed in the fetal liver or any of the adult lymphoid tissues; its expression is limited to kidney, testes, and brain [80]. The importance of Id factors in lymphoid development has been revealed by gene knockout and transgenic studies. Studies involving Id2^−/−^ mice revealed that this factor is essential for the generation of NK cells [81] and DCs [82]. Transgenic expression of Id1 under the control of the *lck* promoter led to a severe block at the DN1 stage of T-cell development [83]. Lastly, Id3 overexpression promoted NK cell development at the expense of T cells [84]. Collectively, these studies have shown that Id factor interference with E-protein activity leads to severe perturbations during lymphoid development.

## 5. E2A and E2-2 in Hematopoiesis

 The functions of E proteins have been most extensively studied in the context of B lymphopoiesis. In B cells, E2A proteins function as homodimers, stabilized by disulfide bonds in a B-cell specific manner [85]. E2A^−/−^ mice lack B cells in fetal liver, bone marrow, and spleen and are prone to die shortly after birth [86]. In the absence of E2A, the early progenitors fail to activate early B-cell developmental genes, such as early B-cell factor (EBF) and the paired box protein 5 (Pax-5), as well as the B-cell specific expression of *Rag* genes [87–89]. As a result, E2A^−/−^ cells fail to undergo *Ig* gene rearrangements and are arrested at the earliest stage of development [88]. In T-cell development, deletion of E2A results in an early partial arrest at the DN1 stage, inappropriate traversal through *β*-selection, and increased positive selection of DP thymocytes (Figure 1(b)) [90–92]. Since E proteins have been shown to compensate for each other [93, 94], studies involving a simultaneous deletion of E2A and HEB were done. These studies revealed defects that were not observed upon deletion of either gene alone. Deletion of E2A and HEB during DN stages revealed a role for E proteins in suppressing proliferation prior to pre-TCR signaling [95]. When both E proteins were deleted in later stages of T-cell development, DP cells developed to the CD8^+^ lineage in the absence of TCR, indicating inappropriate positive selection [96]. In addition, E2A also regulates the expression of *Rag* genes in CLPs [89] and LMPPs [97]. In contrast to E2A, the function of E2-2 is not as well characterized. The most prominent known function of E2-2 is in the regulation of pDC development [98, 99]. In T-cell development, E2-2 has been suggested to play a role at *β*-selection since E2-2^−/−^ mice display an accumulation of DN3 cells (Figure 1(b)) [100].

## 6. HEB in Hematopoiesis

 The importance of HEB factors in lymphopoiesis was revealed by studies involving HEB mutant mice. First, HEB^−/−^ mice were generated by deleting a segment of the bHLH domain, thereby targeting both HEBCan and HEBAlt [93]. In contrast to E2A knockout mice, HEB^−/−^ mice produce B cells, although in reduced numbers [93]. When compared to other E proteins, loss of HEB has the most profound effect on T-cell development (Figure 1(b)). HEB^−/−^ mice have reduced thymic cellularity and display an accumulation of CD8^+^ ISP cells [101], reminiscent of the arrest seen in TCF1^−/−^ and Bcl11b^−/−^ mice. Since the defects observed in HEB^−/−^ thymocytes could not be repaired with anti-CD3 treatment or upon transgenic TCR expression, the functions of HEB were proposed to be either parallel with or downstream of pre-TCR signaling [101]. Moreover, mutant mice expressing HEB without the basic region of the DNA-binding domain render HEBCan and HEBAlt capable of dimerizing but incapable of binding DNA. This dominant negative mutation (HEB^dn^) resulted in a severe block at the DN3 stage of T-cell development [102]. Since T-cell precursors failed to produce V-(D)J rearrangements, HEB was implicated in the regulation of *TCR*β** gene rearrangement. HEB is also involved in the regulation of *pT*α** [103] and *CD4* [104] gene expression, as well as the rearrangement of *TCR*α** gene [105]. However, the relative contributions of HEBAlt and HEBCan to these processes are not well understood.

The arrest at the ISP stage of development in TCF1^−/−^, Bcl11b^−/−^, and HEB^−/−^ mice brings up the question of how these genes are connected, and how they might impact the expression of CD4. We have shown that IL7R signaling is sustained in HEB^−/−^ DN cells [106]. It is, therefore, possible that HEB aids in the downregulation of IL7R signaling after *β*-selection, which is necessary to prevent interference with the upregulation of TCF1, LEF1, and ROR*γ* genes and transition past the ISP stage of development [107]. HEBCan plays an important role in initiation of CD4 gene expression [104], raising the question of whether the CD8^+^ ISP cells in the HEB^−/−^ mice represented DP cells in disguise. However, the cycling profile and intracellular TCR*β* chain expression of these cells suggested otherwise [101]. GATA3 is essential for CD4 gene expression, whereas Runx3 is a direct repressor of CD4 [108, 109]. We found that although HEB deficiency at the DN3 stage did not affect the expression of GATA3, transgenic reconstitution of HEB^−/−^ cells with HEBAlt resulted in the upregulation of CD4 to generate DPs [110]. Therefore, another possibility is that HEB factors, and HEBAlt in particular, function in repressing Runx3 protein expression or activity. This remains to be tested.

Our studies involving the retroviral overexpression of either HEBCan or HEBAlt have shed light on the functions of individual HEB factors in lineage specification and developmental fate decisions. For instance, ectopic overexpression of HEBAlt in LSK cells led to enhanced specification into the T-cell lineage [71] and a reduced capacity to generate myeloid cells [111] in presence of DL1-Notch1 signaling. During B-cell development, HEBAlt overexpression suppressed B-cell potential, even in the absence of DL-Notch1 signals [111]. Lastly, HEBAlt was also shown to play a role in lympho-myeloid specification since precursors with a strong myeloid potential adopted the T-cell fate upon overexpression of HEBAlt [112]. However, the precise mechanisms by which HEBAlt guides T-cell development and fate choice remain to be determined.

In our recent studies, we have shown that HEB^−/−^ mice have an early block in T-cell development, which was alleviated in part upon the addition of an HEBAlt transgene driven by the *lck* promoter. Furthermore, we identified pT*α* and CD3 signaling components as specific targets of HEBAlt during *β*-selection [110]. In addition, HEB^−/−^ mice also had a defect in T-cell commitment, with compromised Notch1 function and a tendency to become DN1-like cells [106]. The DN1-like cells could be induced to differentiate into thymic NK cells, revealing a role for HEB in the T/NK cell lineage decision. Importantly, a new set of interactions were revealed among HEB, Notch1, and GATA3, which regulate the T-cell fate choice in developing thymocytes. Conditional inactivation of either HEBCan or HEBAlt alone will allow for dissociation of their individual functions during T-cell development.

## 7. HEB in the Gene Regulatory Network Controlling the Early T-Cell Development

**Figure 4:**
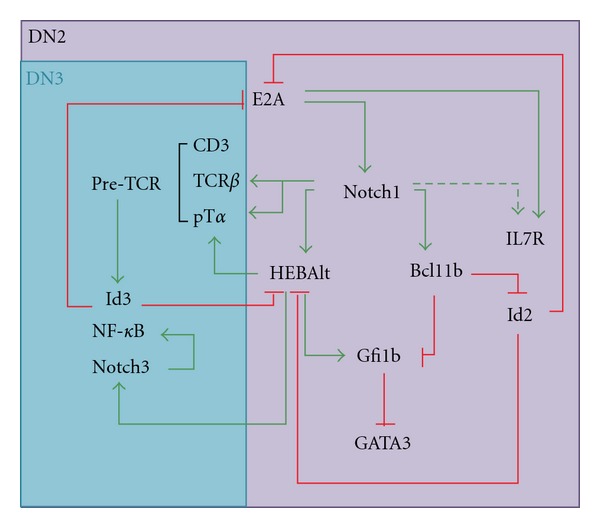
Gene regulatory network model operating in early T-cell development. E2A positively regulates Notch1 expression, which induces the expression of HEBAlt, Bcl11b, and IL7R. HEBAlt positively regulates T-cell genes, such as pT*α* and Notch3, which in turn upregulates NF-*κ*B signaling. Bcl11b negatively regulates Id2 and Gfi1b to balance the expression of GATA3, thus limiting the NK-cell potential. HEBAlt may also regulate GATA3 indirectly through Gfi1b. HEBAlt and Notch1 upregulate pT*α* and TCR*β*, the components of pre-TCR, thus promoting transition from the DN2 to the DN3 stage of T-cell development. Pre-TCR signaling upregulates Id3, which inhibits the activity of E2A and HEBAlt at the *β*-selection checkpoint. The inhibition of HEBAlt activity past the DN3 stage is important as it disrupts the positive feedback loop between Notch3 and NF-*κ*B, which may, otherwise, lead to leukemogenesis. Green arrows show positive inputs, red blunt arrows show negative inputs. Established connections are shown by solid arrows, and indirect or proposed connections are shown by dashed arrows.

The gene networks that operate during early T-cell development integrate developmental regulatory states with the appropriate environmental signals to generate T cells. Although many individual factors have been identified, the connections that exist among them have not yet been well established. Bcl11b, HEBAlt, and TCF1 are positively regulated by Notch signaling in thymic precursors, and both Bcl11b and HEBAlt are sharply upregulated at the DN2a stage of T-cell development, just prior to commitment [47, 54, 71]. Moreover, precursors from both Bcl11b^−/−^ and HEB^−/−^ mice generate NK cells, suggesting that both of these factors are needed to suppress the NK cell fate. Since Bcl11b^−/−^ thymocytes are arrested at the DN2 stage, whereas HEB^−/−^ cells are arrested later in development, it could be proposed that HEBAlt expression is downstream of Bcl11b. However, HEBAlt expression is not considerably reduced in Bcl11b^−/−^ precursors at early stages of development [54]. Likewise, Bcl11b is not reduced in Rag1^−/−^HEB^−/−^ DN3 cells as compared with Rag1^−/−^ DN3 cells (M. Braunstein and M. K. Anderson, unpublished results). Moreover, constitutive Notch signaling did not rescue T-cell development in the absence of HEB [106], indicating that Notch target genes are not sufficient to drive T-cell development in the absence of HEB factors. We, therefore, propose that HEBAlt and Bcl11b function in parallel downstream of Notch signaling to specify the T-cell fate, as illustrated in our gene regulatory network model (Figure 4).

 In early thymocytes, E2A is necessary for the initiation of Notch1 expression, which in turn activates HEBAlt gene expression. TCF1 is also required for the acquisition of the T-cell identity [47]. HEBAlt, therefore, must collaborate with TCF1 to enhance specification to the T-cell lineage, whereas Bcl11b promotes T-cell development indirectly by inhibiting NK-cell development. Indeed, HEBAlt and TCF1 regulate the expression of components of the pre-TCR signaling pathway [47, 110], whereas none of the pre-TCR genes were shown to be affected by the loss of Bcl11b [54]. Together these studies indicate that HEB factors are required for the integration of pre-TCR and Notch signals at *β*-selection and suggest that HEBAlt in particular plays a crucial role in this process.

**Figure 5:**
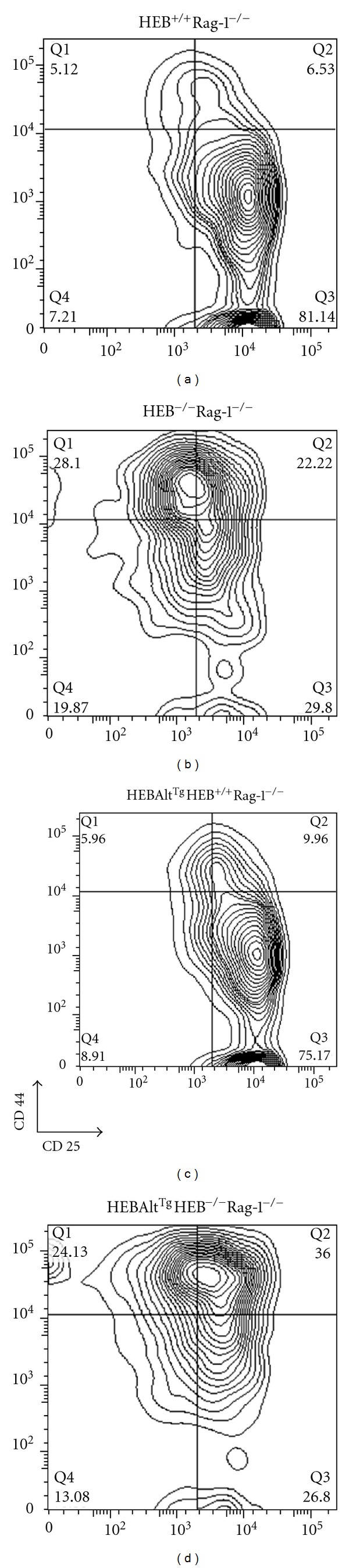
Developmental phenotype of HEB^−/−^Rag-1^−/−^ T-cell progenitors expressing transgenic HEBAlt. HEB^+/−^ mice were bred with Rag-1^−/−^ mice to generate HEB^+/−^Rag-1^−/−^ mice, which were timed mated to generate (a) HEB^+/+^Rag-1^−/−^ and (b) HEB^−/−^Rag-1^−/−^ embryos. Similarly, HEB^+/−^Rag-1^−/−^ mice were bred with HEBAlt^Tg^ mice to generate HEBAlt^Tg^HEB^+/−^Rag-1^−/−^ mice, which were timed mated to generate (c) HEBAlt^Tg^HEB^+/+^Rag-1^−/−^ and (d) HEBAlt^Tg^HEB^−/−^Rag-1^−/−^ embryos. Fetal livers were genotyped, lineage depleted (lineage positive fraction: B cells, myeloid cells, red blood cells). Fetal liver LSK (lineage negative, Sca1^+^, ckit^+^) cells were sorted and cultured on OP9-DL1 for 7 days to allow developmental progression to the DN3 stage. At day 7, lymphocytes were gated on the CD45^+^CD4^−^CD8^−^ fraction and sorted for the DN3 cells (CD44^−^CD25^+^), which were cultured on fresh OP9-DL1 stroma with 5 ng/mL IL7 and Flt3L. Four days later, whole cell cultures were analysed by flow cytometry. All plots were gated on the CD45^+^CD4^−^CD8^−^ fraction.

Although enforced Notch1 signaling was insufficient to support the DN to DP transition in HEB^−/−^ precursors, it was able to effectively restore T-cell potential and suppress NK cell potential in these precursors [106]. It is tempting to speculate that under these conditions it was the induction of Bcl11b by Notch1 signaling that inhibited NK cell development. Bcl11b inhibits the expression of Id2 [54], allowing E2A and HEB factors to maintain the expression of Notch1 and pre-TCR complex genes. GATA3 is negatively regulated by Gfi1b [113]. Therefore, the induction of Gfi1b by E2A and HEB [114] coupled with the repression of Gfi1b by Bcl11b [54] allows fine tuning of the GATA3 levels needed for T-cell development. Our recent results indicate that the transgenic expression of HEBAlt is insufficient to prevent transition into the DN1-like state, consistent with an inability of HEBAlt to upregulate Bcl11b and diversion to the NK cell lineage (Figure 5). Taken together, these studies indicate that HEBAlt and Bcl11b function in parallel during early T-cell development and suggest that whereas Bcl11b inhibits NK and stem-cell gene expression, HEBAlt collaborates with TCF1 to induce T-cell gene expression.

## 8. Life and Death at the *β*-Selection Checkpoint

A lack of HEB gives cells a survival advantage in the absence of DNA rearrangement [106]. Initiation of TCR*β* rearrangements is a key event orchestrating the normal outcomes of *β*-selection. During rearrangement, double-stranded DNA breaks are introduced which, if not repaired, result in death. This removes cells that may otherwise have oncogenic potential. For example, cells that are deficient for the enzyme DNA-dependent protein kinase (DNA-PK) are unable to resolve D-J breaks, which leads to a developmental arrest at the DN3 stage. As a consequence, the cells die via a p53-dependent pathway. On the other hand, T-cell progenitors that do not express *Rag* genes are unable to initiate DNA breaks during D-J rearrangement. Indeed, these cells have very low amounts of p53 compared to cells that are DNA-PK deficient, indicating that they escape death initiated by the p53-dependent pathway. However, Rag-deficient T-cell precursors still die. Although the mechanism of death has yet to be determined, it is likely to involve a combination of events that include upregulation of proapoptotic molecules, such as Bim, by the FOXO factors and the absence of pro-survival signals that emanate from the pre-TCR, Notch1 and IL7 signaling pathways [115]. Both pre-TCR and IL7R signal via PI3K, which inhibits the activity of FOXO factors [116]. Bim is also upregulated directly by E2A [115] and could be a direct target of HEB as well. In one scenario, accumulation of E proteins in DN3 cells that lack TCR*β* rearrangements would result in upregulation of Bim and elimination by apoptosis. Interestingly, Notch1 signaling also mediates survival via Akt, not only in normal DN3 cells but also in Rag-deficient DN3 cells [117].

HEBCan and E2A factors suppress proliferation by upregulating cell-cycle inhibitors [118], which normally keep DN3 cells without rearrangements in check. Interestingly, an alternative outcome was available to certain HEB^−/−^ DN3 cells at the time of *β*-selection: development into the thymic NK cell lineage. Although HEB^−/−^ T-cell precursors with rearranged TCR*β* genes and intact Notch1 signaling had the ability to turn into DN1-like cells, the majority of the cells that became DN1-like lacked TCR*β* rearrangements and had downregulated Notch signaling. Even though restoring full Notch signaling did not restore the ability to pass through *β*-selection in the absence of the pre-TCR, it did restore the natural outcome of DN3 cells without rearrangements: death. The mechanism by which Notch signaling could overcome HEB deficiency to induce death is unknown. The tumour-suppressive function of E2A [119] and likely HEBCan is in contrast with the activity of HEBAlt, as we have observed that HEBAlt transgenic mice develop lymphoma, possibly through sustained Notch1 signaling (M. Braunstein and M. K. Anderson, unpublished results). Under normal circumstances, both HEBAlt and Notch1 are downregulated at *β*-selection. In the transgenic mice, however, Id3 was likely insufficient to block the activity of HEBAlt, which might have led to lymphomagenesis by maintaining Notch1 signaling across the *β*-selection checkpoint.

## 9. Development of T versus tNK Cells in the Thymus

From an evolutionary standpoint, Notch signaling is an ancient pathway, whereas pre-TCR signaling is a relatively new acquisition. The NK cell gene program, therefore, may represent a default route for early progenitors in the ancient thymus, which later in evolution became circumvented to generate cells with rearranged receptors. Indeed, NK cells are generated first in the fetal thymus prior to any *αβ* T cells [120]. Moreover, the requirements for GATA3 and IL7R are common between T cells and thymic NK cells, and while the development of thymic NK cells may not depend on Notch1 signaling [121], evidence for a role of Notch in thymic NK cell development does exist [122]. Therefore, the evolutionary divergence of the thymic NK and T-cell lineages may be mirrored by the developmental steps that give rise to each lineage. HEB may in part be responsible for the separation of these lineages, by modulating Notch signaling and selective survival of *β*-selected T-cell progenitors, and by regulating the levels of GATA3.

**Figure 6:**
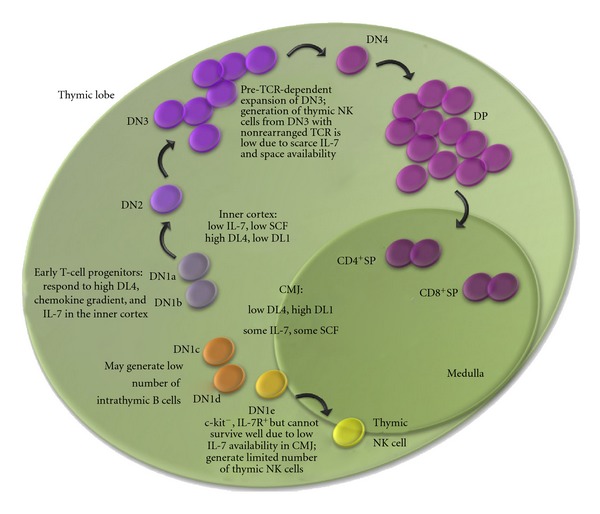
Development of T versus tNK cells in the thymus. DN1 cells enter thymus at the corticomedullary junction (CMJ). This area is low in DL4 but high in DL1 ligand; in addition, there is a low amount of IL-7 and SCF dispersed throughout a thymus. DN1c and DN1d cells may be progenitors to a small number of B cells generated intrathymically. DN1a and DN1b cells represent canonical early T-cell progenitors (ETPs), which migrate to the inner cortex, the area of high DL4 ligand concentration. In response to Notch signalling, ETPs turn on many of the T-lineage specific genes and develop into DN2 cells. At the DN3 stage, the cells rearrange TCR*β* genes and undergo *β*-selection, thus expanding and taking up most of the space in the outer cortex. This is disadvantageous for those DN3 cells that have not rearranged TCR*β*, which may give rise to the thymic NK cells. Thus, a small percentage of NK cells may be generated in a thymus, mostly from the DN1e progenitors, which are likely to remain near the CMJ region where the DL4 ligands are low and the IL-7 concentration is sufficient for the thymic NK cell development.

In our studies, thymic NK cells were derived from HEB^−/−^ DN3 cells that would not have survived or developed in the absence of Notch signaling, suggesting an initial role for Notch in specifying a common T/NK progenitor. It is also possible that tNK cells normally arise from noncanonical precursors such as DN1c, DN1d, or DN1e cells. DN1e cells are of particular interest because they already express high levels of IL7R and Id2 [4, 6]. Although they do not have strong proliferative potential, they do generate both T and NK cells [4]. Interestingly, culturing DN1e cells on OP9 stroma that lacks DL expression yielded only 3% NK cells, whereas culturing ETPs on OP9 stroma generated approximately 40% NK cells. This raises the intriguing possibility that DN1e cells are primed to become thymic NK cells but need intermittent and/or low DL-Notch signals to give rise to thymic NK cells (Figure 6). Consistent and/or high DL-Notch1 signaling, on the other hand, would be expected to promote noncanonical T-cell development from DN1e cells.

The DN1 and DN2 stages of T-cell development express many progenitor-like genes [123, 124] that allows for their experimental reprogramming into mast cells and NK cells. Under normal conditions, however, the DN3 stage marks the point of no return; at this stage, the cells either commit to the T-lineage or die. The question then arises: what defines the DN3 stage and T-cell commitment? Development to the DN3 stage does not require the rearrangement of TCR*β* genes or the expression of *Rag* genes, as indicated by the ability Rag-1^−/−^ thymocytes to acquire the T-lineage phenotype up to this stage. Instead, the upregulation of many other T-cell specification genes must be used as the criteria to determine the developmental status of an early T-cell progenitor. Commitment, on the other hand, is defined as the inability to adopt alternative lineage choices. HEB^−/−^ DN3 cells display an interesting and aberrant gene expression pattern that speaks to these criteria: they have a partially activated T-cell program, and they maintain a limited ability to differentiate into an alternative fate. Therefore, it is unlikely that the HEB-deficient DN3 cells, which can give rise to thymic NK cells, reflect DN2 cells in disguise. Rather, the transition from the DN3 to DN1-like state involves a true loss of T-cell identity in the absence of cell death.

The thymus provides a highly structured and ordered environment, where Notch ligands and cytokines become available in varying doses and in specific niches, tightly controlling cellular development [125]. These restrictions promote early T-cell development and limit the selection of both DN3 cells that lack TCR*β* rearrangements and thymic NK cell development. A T-cell progenitor entering the thymus through the CMJ is exposed to the DL1 ligand and SCF, but low IL-7 availability (Figure 6). At this point, progenitors such as DN1e cells, which are c-kit^−^ and IL7R^+^, could potentially respond to DL1 but would be limited in their survival and thus fail to generate abundant thymic NK cells. The distribution and levels of IL15 within the thymus still need to be determined; however, it is likely that IL15 is only scarcely available throughout the thymus given the small number of thymic NK cells that are generated even in a Rag-deficient thymus. Lastly, the expression of chemokine receptors on DN1e cells suggests that these cells may migrate towards the medulla rather than the cortex, which could provide an alternative set of signals that would promote NK cell development [6]. ETPs, on the other hand, lack IL7R but express c-kit and chemokine receptors that would help with transition from the CMJ to the cortical region. In the cortex, the rapid expansion of *β*-selected cells allows the T-cell precursors to outcompete and thus limit the survival and developmental capacity of DN3 cells lacking TCR*β* rearrangements. Thymic epithelial cells express abundant levels of IL7 throughout the entire fetal thymus from day E12.5 to E13.5 [126]. Therefore, the availability of IL7 within an E12.5–E13.5 fetal thymus would be expected to encourage thymic NK cell development. Indeed, thymic NK cells develop in the fetal thymus before any DP cells are generated. After E15.5, however, the thymus size increases due to proliferating thymocytes and the proportion of epithelial cells producing IL7 is correspondingly reduced. Approximately 15% of the cells in fetal thymic organ culture are thymic NK cells, whereas adult Rag-deficient thymus contains approximately 4% thymic NK cells. By contrast, thymic NK cells represent only 0.013% of the adult thymocyte population. Our results showed that, although HEB^−/−^ precursors had downregulated Notch signaling and indeed gained DL independence, they were nonetheless still dependent on IL7 to survive [106].

## 10. Summary

In summary, HEB factors are essential mediators of T-cell lineage specification and commitment. HEBAlt and HEBCan play distinct roles in these processes, with HEBAlt inducing T-lineage genes and suppressing myelopoiesis within the thymus, whereas HEBCan appears to be more involved in repressing the NK cell fate. These factors interface with Notch1, TCF1, GATA3, Bcl11b, and Gfi1b to form a network of interactions that not only initiates the T-cell program but also incorporates positive feedback loops that sustain it. Further study will be needed to address the question of how HEBAlt and HEBCan function as homodimers, heterodimers with each other, or heterodimers with E2A, but our work has clearly shown that both HEBCan and HEBAlt are central factors in the early stages of T-cell development.
